# A FeAsibility items Checklist for assessing implementation characTeristics of patient-reported Outcome measures in Research, Regulation and Routine clinical care (FACTOR3): Development and evaluation

**DOI:** 10.1016/j.clinme.2026.100592

**Published:** 2026-05-27

**Authors:** Asad Bhatty, Chris Wilkinson, Visvesh Jeyalan, Ali Wahab, Jeremy Dwight, Marcin Ruciński, Edwin de Beurs, Melanie Calvert, Richard P. Gale, Mike Horton, Andrea Manca, Tom Melvin, Massimo Di Maio, Amar Rangan, Julie Sanders, Tonya Winders, Adam B. Smith, Chris P. Gale, on behalf of the FACTOR3 Consortium

**Affiliations:** a Leeds Institute of Cardiovascular and Metabolic Medicine, University of Leeds, Leeds, UK; b Leeds Institute for Data Analytics, University of Leeds, Leeds, UK; c Department of Cardiology, Leeds Teaching Hospitals NHS Trust, Leeds, UK; d Hull York Medical School, University of York, York, UK; e Academic Cardiovascular Unit, South Tees NHS Foundation Trust, James Cook University Hospital, Middlesbrough, UK; f European Society of Cardiology Patient Forum, UK; g Department of Clinical Psychology, Leiden University, Netherlands and Arkin Mental Health Institute, Amsterdam, the Netherlands; h Centre for Patient-Reported Outcome Research (CPROR), Department of Applied Health Sciences, University of Birmingham, Birmingham, UK; i National Institute of Health and Care Research (NIHR) Blood and Transplant Research Unit (BTRU) in Precision Cellular Therapeutics at the University of Birmingham, UK; j NIHR Applied Research Collaboration (ARC) West Midlands, Birmingham, UK; k Birmingham Health Partners Centre for Regulatory Science and Innovation, University of Birmingham, Birmingham, UK; l York and Scarborough Teaching Hospitals NHS Foundation Trust, York, UK; m Psychometric Laboratory for Health Sciences, University of Leeds, Leeds, UK; n Centre for Health Economics, University of York, Heslington, York, UK; o School of Medicine, Trinity College, University of Dublin, Dublin, Ireland; p Department of Oncology, University of Turin, Division of Medical Oncology, Ordine Mauriziano Hospital, Turin, Italy; q Academic Centre for Surgery, The James Cook University Hospital, Middlesbrough, UK; r Faculty of Nursing, Midwifery & Palliative Care, King's College London, London, UK; s Global Allergy and Airways Patient Platform, Vienna, Austria

**Keywords:** Patient-reported outcome measures, PROMs, Feasibility, Implementation

## Abstract

**Study objective:**

Patient-reported outcome measures (PROMs) provide valuable data to inform regulatory decision making, health technology assessment and routine clinical care. We aimed to develop a feasibility item checklist for PROMs and their selection, beyond their psychometric properties (FACTOR3).

**Methods:**

We followed a five-stage method to select parameters for PROM evaluation. 1) A scoping literature review identified candidate items for consideration; 2) round 1 modified Delphi was used to select items for inclusion or exclusion, conducted by the design group (n = 14); 3) feedback on the checklist was provided by representatives from the European Medicines Agency and National Institute for Health and Care Excellence; 4) round 2 modified Delphi was used to finalise item selection, conducted by the clinical domains group (n = 21) and 5) evaluation of the feasibility item checklist using a selection of PROMS (EQ5D-5L, HeartQOL, the Oxford Hip Score, The EORTC Core Questionnaire QLQ-C30, Re-QOL-10 and NEI-VFQ-25) (Fig. 1).

**Results:**

The scoping review identified 13 items relating to the intrinsic feasibility of using PROMs which were considered in the modified Delphi. The final FACTOR3 checklist included eight unique candidate items: price; licensing, comprehensibility, duration, coverage, translations, electronic device compatibility; and minimal important difference. Of the six PROMS evaluated, the intraclass correlation coefficient was 0.81, suggesting good reliability.

**Conclusions:**

FACTOR3 is a feasibility item checklist to assess the implementation characteristics of PROMs in research, regulation and routine clinical care. It may be used in conjunction with existing psychometric evaluation and user guides for PROMS to facilitate their use in health care.

## Highlights

• There are additional feasibility factors intrinsic to a PROM, beyond its psychometric properties, that impede its routine use
• Eight variables were selected through consensus of interdisciplinary stakeholders that was agreed upon by European regulators
• The FACTOR3 checklist evaluates a PROM's feasibility characteristics with broad applicability in clinical care and trials.
• It may be used with psychometric evaluation thus providing a comprehensive selection process for contemporary use of PROMs

## Introduction

Patient-reported outcome measures (PROMs) provide valuable information on the impact of disease and treatment on a patient’s symptoms, emotional wellbeing, functioning and quality of life.^1^, ^2^, ^3^ Trial data on PROMs provide evidence of the efficacy and tolerability of treatments from the patient perspective and are valued by regulators.^4^, ^5^ PROMs are increasingly used in routine clinical practice and may be used to support patient communication, shared decision making^6^ and early detection of treatment side effects.^7^, ^8^

The European Medicines Agency (EMA) and the US Food and Drug Authority recommend the use of PROMs for medical device or drug evaluation, recommend that such PROMs be validated in the target population and disease, and the results appropriately communicated to healthcare providers and patients when supporting pharmaceutical labelling claims.^4^, ^5^ In the UK NHS, PROMs are routinely used to evaluate the outcomes of elective hip and knee surgery, with results informing an assessment of healthcare provider performance, as well as supporting clinical decision making^9^ and benchmarking quality of care.^10^, ^11^, ^12^

There are over 7,000 validated PROMs spanning the range of medical diseases and interventions,^13^ but limited guidance exists in formally assessing their intrinsic feasibility of implementation. The Patient-Reported Outcomes Tools: Engaging Users and Stakeholders (PROTEUS) consortium recommends a qualitative assessment of the reliability and validity of a PROM prior to its selection,^14^ such as The ‘COnsensus-based Standards for the selection of health Measurement INstruments’ (COSMIN) evaluation.^15^ Assessment of some feasibility items is recommended, such as the cost of using a PROM, licensing^16^, ^17^, ^18^ and the ability of a PROM to be understood by the user.^19^ However, there are additional factors such as operational costs, including searching for more information on a PROM through subscription websites,^13^ regulatory approval for the use of a PROM in a randomised clinical trial^20^, ^21^ and metrics that interpret change in PROM scores over time, such as the minimal important difference (MID) that determines the ability of a PROM to be used in research, regulatory affairs and routine clinical care,^22^, ^23^ but are not included in recommended assessment guides.

We aimed to develop a feasibility item checklist for assessing the implementation characteristics of PROMs in research, regulatory affairs and routine clinical care, and evaluate it against commonly used PROMs. This could complement user guides and address an implementation gap (Fig. 2).

## Material and methods

The development of the FACTOR3 checklist consisted of five stages: 1) scoping review to identify candidate items for consideration, conducted by the steering committee; 2) first stage modified Delphi to include or exclude items, conducted by the design group; 3) feedback on the checklist by the regulator group; 4) second stage modified Delphi to provide feedback on items, conducted by the clinical domain group; and 5) evaluation of the feasibility item checklist using a selection of PROMS.

### Steering committee

The steering committee comprised clinical experts (A.B., C.W., C.P.G), a PROMs methodologist (A.B.S.) and a data manager (S.C.). They designed the study, formulated a list of candidate variables from the scoping review, identified and invited individuals to the other three working groups (design, clinical domain and regulator group), and collated and presented the results of the polls to the groups.

### Scoping review

A scoping review was performed by the steering committee to identify potential factors in the published literature and existing user guides. All prospective and retrospective articles and grey literature^2^, ^14^, ^24^ relating to feasibility of implementation of PROMs in adults from inception to 6 March 2024 in English was included. PubMed was searched using a pre-defined search strategy (Supplementary Section) and was conducted by A.B. These were assimilated and formed the basis of the questions for voting.

### Modified Delphi

The steering committee identified potential members for each of the working groups by selecting co-authors of PROM research manuscripts, those holding senior positions in regulatory affairs, healthcare professionals with experience of PROMs, and from personal references and recommendations by other Delphi participants. The aim was to achieve wide representation across multiple geographies (Europe, North America and Oceania) and clinical and academic disciplines. We therefore contacted PROM methodologists, developers of PROMs, health economists, biostatisticians, patients, patient representatives and multidisciplinary healthcare providers. No minimum sample size was placed on the number of working group members and Delphi participation. Each working group member’s expertise was confirmed by the steering committee (apart from patients and patient representatives) before being invited to participate via email. Once accepted, the steering committee allocated each panellist either the design or clinical domain group detailed below, sent them an information pack via email and invited them to attend a virtual presentation about the project.

The first stage of modified Delphi meetings consisted of 14 experts as part of the design group who voted in the online survey. Participants were invited to provide feedback via free text, during voting and discussion was encouraged around the rationale for including/excluding items distilled from the scoping review and the scope of the checklist. Feedback to provide additional items was welcomed.

Once the list of variables and their definitions for the checklist were agreed, they were reviewed by the regulators for their feedback and agreement. The group comprised of representatives from the European Medicines Agency (EMA) and the National Institute for Health and Care Excellence (NICE) who were either PROM methodologists, patient representatives or trialists. They did not participate in the voting process.

The second stage of modified Delphi meetings consisted of 21 experts as part of the clinical domain group who voted in the online survey from the results garnered from the first stage Delphi. Participants were encouraged to include/exclude items distilled from the design group. Members of the design group and steering committee only voted once in the first stage to prevent unequal weighting of votes.

The virtual presentation was delivered and chaired by A.B. and supported by C.W. and C.P.G. and sought to summarise the current PROM selection process as per user guides, highlight aspects of feasibility that are not addressed in the literature, and the candidate feasibility items extracted from the scoping review for discussion. Following the presentation, panellists were emailed an online survey. All panellists received the same presentation and options for voting.

The survey was in English using the ‘Jisc’ platform (https://www.jisc.ac.uk/). Participants were asked to provide their name and email address to protect against duplicate voting. As part of the survey, participants were asked to review the candidate feasibility item checklist variables that had been generated from the scoping review and to vote to ‘include’ or ‘exclude’ each variable. Judgement was based upon the panellist’s perception of the feasibility and applicability of each variable. Written comments were invited for each item, which were collated and thematically categorised by the steering committee.

**Fig. 1:**
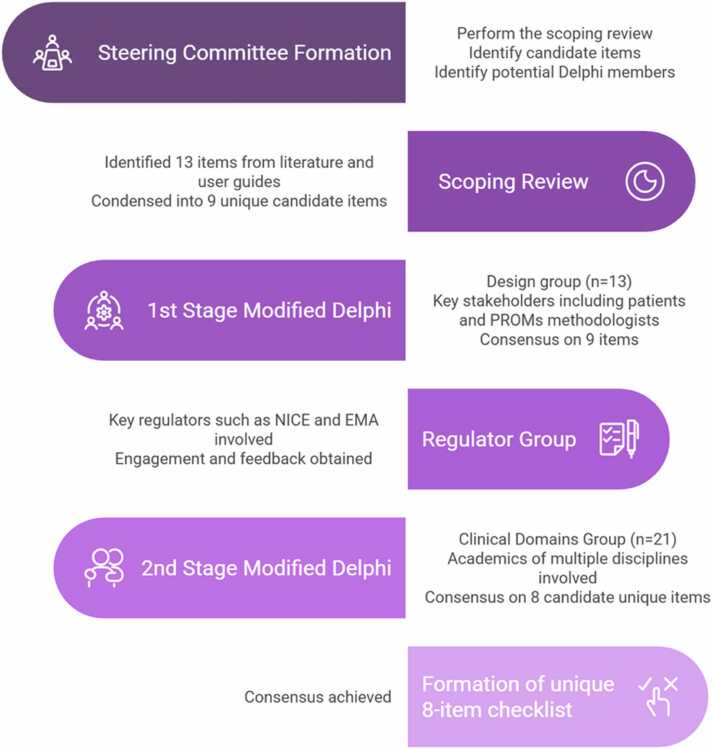
Visual graphic on the formation of the FACTOR3 checklist.

The threshold for inclusion of a candidate feasibility item checklist variable was at least 75% of panellists voting on an item to include and was decided *a priori*.^25^, ^26^ The threshold for exclusion was at least 75% of participants selecting for the variable to be excluded or achieving less than 75% to include. No financial or in-kind incentive was offered to panellists for completing their survey or virtual meeting. The overall process is shown in Fig. 1.

**Fig. 2:**
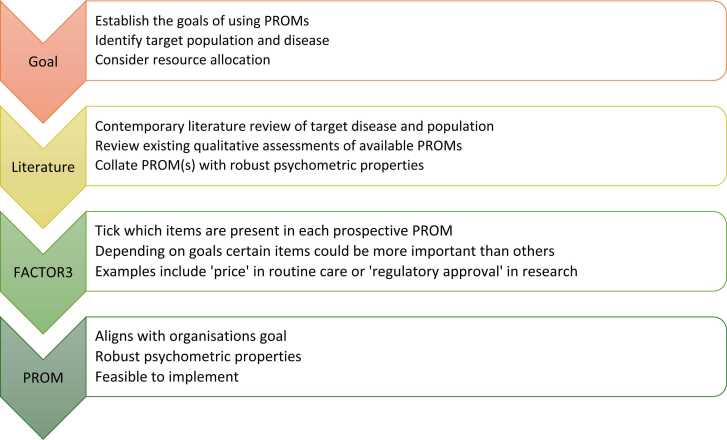
Visual graphic on how the FACTOR3 checklist fits into the selection process.

### Evaluation of checklist

Three clinician reviewers who were independent of the modified Delphi method for the development of the feasibility item checklist, were invited to evaluate six PROMs against the final checklist. Specific PROMs (EQ5D-5L, HeartQOL, the Oxford Hip Score, The EORTC Core Questionnaire QLQ-C30, Re-QOL-10 and NEI Visual Function Questionnaire-25) were selected because they were routinely used in clinical practice (for example within the NHS of England), endorsed by international organisations, widely cited in the literature and to cover different clinical specialties. Each reviewer was given a sample of the questionnaires and URL links to each organisation’s website, as some checklist items may be difficult to answer on a questionnaire alone. For example, licensing and cost information will not be available by reviewing the questions in a PROM.

The presence of each item of the feasibility item checklist was assigned a score of 1 and the overall score was recorded. A PROM that scored highly against the feasibility item checklist was deemed feasible or easy to implement, with feasibility being defined as the intrinsic characteristics of a PROM that allows ease of implementation. This differs to alternative definitions of feasibility that assess the extent to which a PROM may be successfully used or carried out within a particular setting.^27^ The intraclass correlation coefficient score (ICC) was performed to evaluate reviewers’ inter-rater reliability, which was collated and stored in MS Excel.

### Patient and public involvement

Two patient representatives from the European Society of Cardiology Patient Forum (J.D. and M.R.) were involved in the first stage modified Delphi meetings and one patient representative (T.W.) was involved in the second stage modified Delphi meetings. They provided feedback on all candidate items extracted from the steering committee and scoping review and emphasised the inclusion of patient comprehension and comprehensiveness of a PROM into the checklist. These two items achieved consensus.

## Results

### Steering committee

Following the scoping review, an initial list of 13 items was drafted by A.B. (see Supplementary Table 1). The steering group reviewed these and merged some items together to form a list of nine unique candidates. This list was used in the modified Delphi exercise.

### Design group

**Fig. 3:**
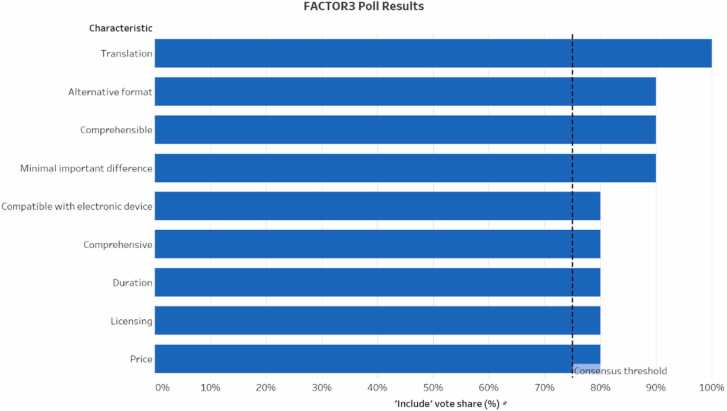
Voting distribution of first stage modified Delphi with the design group.

This consisted of five PROM methodologists, one biostatistician, two health economists, four healthcare providers and two patient representatives. All participants were from Europe (n = 14, mostly the UK), and there were nine men and five women. A series of engagement and feedback meetings was held, with subsequent voting on the nine items. The results were then presented and discussed among the design group during an online meeting, and the design group voted to include all nine items. The distribution of votes is shown in Fig. 3. There was detailed discussion about the ideal scope of the checklist, whether additional variables were required and whether there should be weighting of items to give more importance to some items over others.

### Clinical domain group

**Fig. 4:**
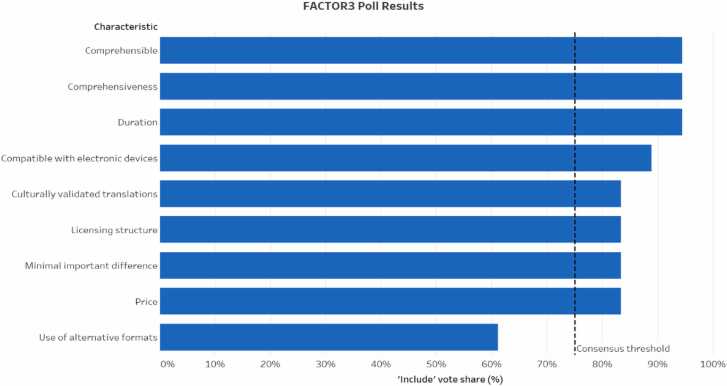
Voting distribution of second stage modified Delphi with clinical domains group.

The group included individuals with expertise in cardiology (n = 4), ophthalmology (n = 3), dermatology (n = 3), orthopaedics and rheumatology (n = 3), medical oncology (n = 2), mental health (n = 1), neurology (n = 1), clinical epidemiology (n = 1), urology (n = 1), medical device regulatory affairs (n = 1), and a patient representative (n = 1). The experts were from Europe (n = 12), North America (n = 7) and Oceania (n = 2) and comprised 14 men and seven women. Sequential meetings were held amongst the 21 experts from the clinical domain group. From the nine-candidate list of items derived from the scoping review and design group consensus, eight items were voted on to include into the FACTOR3 checklist. The distribution of votes is shown in Fig. 4. There was consistent feedback over the scope of the checklist, refinement of the names of items and how to utilise the checklist in both trials and clinical practice, which is reported below.

### FACTOR3 checklist

The final feasibility items checklist (FACTOR3) includes eight items and is to be used in addition to the psychometric evaluation of a PROM. The checklist is provided in the Supplementary Section.

1) *Licensing structure that aligns with the goal*
  Often validated PROMs are subject to licensing arrangements that may vary according to intended use, and obtaining appropriate permissions can be time consuming and possibly subject to prohibitive terms and conditions involving access to data. Panellists felt that these were important considerations during a PROM selection process.^17^ Some panellists also cautioned against the use of unlicensed PROMs because it raised uncertainty over the origin of a questionnaire and if any results obtained would be valid.
2) *Cost*
  This domain refers to both using a PROM and its operational cost. Linked to the licensing structure of a PROM is the cost of administering and managing it, which may impede its routine use.
  Operational costs that may be incurred involve implementing an electronic PROM into existing software of electronic health records. Also, obtaining a valid and culturally appropriate translation could incur costs; the panellists felt that this may be a barrier for some organisations and should therefore be considered when selecting a PROM.^28^, ^29^
3) *Comprehensibility*
  Results from PROM data may be unreliable if patients refuse to complete the questionnaires because they do not understand the questions (leading to increased missingness of data) or, worse, answer while not fully comprehending the question.^30^ This problem is compounded if the PROM was developed with no or limited patient involvement.^31^ Allowing the content of a PROM to be at a grade six or age 10 reading level can help ensure patient comprehension as literacy levels vary across the population, and increases the likelihood of patients completing the questionnaire.^29^ Although comprehension overlaps with content validity, and therefore assessed in qualitative assessments,^15^ our patient representatives emphasised its inclusion over others. Similar to other items, our panellists recommended the use of a patient group reviewing the comprehensibility of potential PROMs prior to their implementation.
4) *Translated and validated into the language of choice*
  Another way that PROM data could be unreliable is if there is no validated translation that isw culturally adapted to provide equivalent meaning to the original PROM for a diverse patient population.^29^ This was agreed to and is recommended by regulators.^32^
5) *Duration*
  Longer PROMs are associated with low respondent rates in comparison to shorter ones, especially for patients who are ill or fatigue easily.^21^ However, PROMS that are long and/or take longer to complete can be important, depending on the target population and objective of data collection objectives. Participants may be willing to complete lengthier measures if they understand why the data are being collected and how they will be used.^33^ The panellists agreed that the choice of PROM should be considered against the duration of time it takes for completion, as well as the organisation’s goal in using the PROM. Some panellists advised for a local patient and public group to be involved early in a project to determine if the PROM has a high respondent burden or not.
6) *Comprehensiveness*
  This item had two considerations. The first was whether the PROM had regulatory approval to be used as an outcome measure in the targeted area of research. The panellists felt that regulatory approval was equally as important as having robust psychometric properties.
  The second consideration was how well the PROM encapsulates the elements of the target disease. Striking the balance between shorter questionnaires and adequate coverage that doesn’t contribute to respondent burden was also raised. Comprehensive PROM coverage improves the reliability of responses by ensuring PROM data reflects the patient perspective entirely.^34^ The experts stressed its inclusion despite some overlap with a questionnaires content validity^35^ and therefore psychometric evaluation. The experts emphasised that the PROM should be validated in the target population.
7) *Electronic device compatibility*
  Many healthcare organisations use electronic health records, and the ability to incorporate a PROM with its software or complete it by smartphone use is clearly advantageous. This increases response rates and allows questionnaires to be completed beyond the hospital visit.^36^ Subsequent interpretation and analysis of data is less burdensome than using paper records. The ability to implement an electronic questionnaire streamlined with existing healthcare systems that is appropriate and acceptable to licensed owners of the PROM was also a consideration. Some panellists felt that in some circumstances a PROM may be compatible with a smartphone app but not incorporated with healthcare records, which may conversely increase administrative burden.
8) *Minimal important difference*
  The MID provides a clinical context to the results of a PROM and is therefore meaningful to clinicians. It provides a measure of the smallest change in the PROM of interest that patients perceive as important, either beneficial or harmful, and that may potentially warrant a change in management.^22^ A barrier to PROM implementation is negative clinician perspectives,^8^ and the panellists felt that providing appropriate context to PROM results was, therefore, important.

### Factors not included in the final checklist

One item that was not included in the final checklist was the availability of the PROM to be provided in an alternative format. Alternative PROM formats give a method of improving the reliability of responses. These include visual analogue scales of PROMs^37^ to improve comprehension and reduce respondent burden. Furthermore, a concern from focus group members was to ensure adequate support for all patient populations to complete an assessment such as, but not limited to, patients with visual or hearing impairment and neurodivergent patients.

### Checklist evaluation

The results of each reviewer’s assessment of the PROMs are in Supplementary Table 2. The calculated ICC was 0.81 suggesting the checklist has good inter-rater reliability.

## How to use the FACTOR3 checklist

The FACTOR3 checklist evaluates the intrinsic feasibility characteristics of a questionnaire and can streamline the PROM selection process prior to implementation in research, regulatory affairs and routine clinical care. The PROTEUS guide remains an important document to consult that elaborates the current selection process; identifying all available questionnaires on a disease domain and evaluating their psychometric properties.^2^, ^14^ We recommend completing the FACTOR3 checklist early in the PROM selection process, such as after the psychometric evaluation stage. This ensures that all important feasibility concerns are comprehensively addressed prior to implementation that aligns with the organisation’s goals.

These goals could differ depending on available resources, target population characteristics and context of care. For example, in research, comprehensiveness in terms of regulatory approval could matter more to an organisation than other items and help to decide which PROM to choose further on top of its qualitative assessment. Whereas other items such as price and licensing structure of a questionnaire for routine clinical care in a publicly funded institution may be paramount. Therefore, the Delphi panellists voted against weighting each item which allows the checklist to be flexible with any context.

## Discussion

Using a modified Delphi approach that was inclusive of a scoping review, expert opinion and stakeholder involvement, we developed and evaluated the FACTOR3 feasibility item checklist for assessing the intrinsic implementation characteristics of PROMs in research, regulation and routine clinical care. FACTOR3 may be used in conjunction with existing psychometric evaluation,^15^ regulatory guidance,^4^, ^38^ ethical^39^ and protocol guidance,^2^ PROTEUS trials and practice resources^14^, ^24^, ^40^ and other user guides for PROMs – providing a comprehensive and complementary selection process for the contemporary selection of PROMs. FACTOR3 has eight components – price, licensing, comprehensibility, duration, translation, coverage, electronic device compatibility and minimal important difference, which were informed by the literature and international experts and stakeholders in PROMs. Our evaluation of six PROMs (frequently employed in research and or clinical settings) against FACTOR3 criteria by three independent assessors found that the feasibility item checklist provided consistency in results.

Generating high-quality patient-reported outcome data informs clinical care and the generalisability of trial results yet is affected by respondent burden of the target population.^3^, ^19^, ^21^, ^41^ High levels of missing data or unreliable responses due to poor comprehension can lead to misinterpretation of results.^2^ This can deepen health inequalities in poorly served populations, partially due to feasibility concerns.^42^ The lack of available valid translations, and poor comprehension and digital literacy, impact underserved communities that are traditionally underrepresented in research and trials the most.^41^, ^43^ Therefore the Delphi panellists felt that addressing respondent burden in more depth in a feasibility checklist was needed, which differs from the PROTEUS guide.^33^ Maximising the response rates and reliability needs to be catered to the diverse patient population, which differs by socioeconomic status and literacy.^41^, ^44^ PROMs that are available in electronic formats, as well as the availability of pen and paper options, may suit patients with poorer digital literacy who do not own an electronic device.^45^ However, the use of electronic health records is widespread and the ability of a PROM to align with existing software may improve compliance,^36^ provides a source for data collection and storage^46^ and is some patients’ preferred mode of completing questionnaires.^41^ All these factors could be implemented without bias, as a meta-analysis into mixed modes of administration of PROMs has shown.^47^

To our knowledge, this checklist is the first to evaluate the intrinsic feasibility of implementing a PROM with broad applicability for clinical practice and trials. It provides a practical supplement to existing psychometric and quality assessments in the evaluation of prospective PROMs depending on the organisation’s goals that is in line with regulatory requirements.^4^, ^38^ We identified a list of intrinsic feasibility items necessary for consideration prior to the selection and adoption of PROMs distilled from expert opinion using the modified Delphi method. However, we recognise limitations of this work. We used a focused definition of feasibility, whereas other more broad definitions exist such that evaluate the extent to which a PROM may be successfully carried out within a particular healthcare or research setting.^27^ Furthermore, to ascertain some aspects of feasibility (such as licensing and cost) requires users to explore a diverse range of materials to clarify which may increase administrative burden initially.^17^ Although the items were filtered from a scoping review, the final checklist was agreed upon by consensus of experts and is therefore subject to potential selection bias. Furthermore, the group of experts and stakeholders were chosen to be a representative sample, but the sample size was small and completed in English only. However, the experts had broad expertise in PROMs use in clinical practice and trials, and the stakeholder group consisted of major European regulators and patient representatives that shaped the discussion and provided further strength to our findings.

## Conclusion

Informed by scoping review, and modified Delphi methods including PROM methodologists, clinicians, patients and regulators, we have developed a checklist of implementation feasibility items that should be considered when selecting a PROM. We anticipate that the keys users and beneficiaries of FACTOR3 will be researchers conducting studies and writing papers, the healthcare sector when considering a PROM for routine use and assessment of clinical practice, and regulators for the evaluation the success of clinical outcomes in regulatory decision making. The checklist could have broad applicability and serves to act as a decision-making tool, in conjunction with existing psychometric assessments and user guides, for the selection of a PROM.

## CRediT authorship contribution statement

**Tom Melvin:** Writing – review & editing, Methodology, Investigation. **Asad Bhatty:** Writing – review & editing, Writing – original draft, Visualization, Investigation, Formal analysis, Data curation. **Massimo Di Maio:** Writing – review & editing, Methodology, Investigation. **Chris Wilkinson:** Writing – review & editing, Supervision, Methodology, Investigation, Formal analysis. **Mike Horton:** Writing – review & editing, Methodology, Investigation. **Andrea Manca:** Writing – review & editing, Investigation. **Tonya Winders:** Writing – review & editing, Methodology, Investigation. **Jeremy Dwight:** Writing – review & editing, Project administration, Methodology, Investigation. **Adam B. Smith:** Writing – review & editing, Supervision, Software, Resources, Formal analysis, Data curation. **Marcin Ruciński:** Project administration, Methodology, Investigation. **Amar Rangan:** Writing – review & editing, Methodology, Investigation. **Visvesh Jeyalan:** Writing – review & editing, Validation, Project administration. **Julie Sanders:** Writing – review & editing, Validation, Resources, Methodology, Investigation, Formal analysis. **Ali Wahab:** Writing – review & editing, Validation, Data curation. **Richard P. Gale:** Writing – review & editing, Resources, Investigation. **Chris P. Gale:** Writing – review & editing, Supervision, Resources, Project administration, Funding acquisition, Formal analysis, Conceptualization. **Edwin de Beurs:** Writing – review & editing, Validation, Methodology, Investigation. **Melanie Calvert:** Writing – review & editing, Supervision, Methodology, Formal analysis.

## Ethics approval and consent to participate

No ethical approval was required for this scoping review and modified Delphi exercise.

## Funding

This research did not receive any specific grant from funding agencies in the public, commercial, or not-for-profit sectors**.**

## Declaration of competing interest

The authors declare the following financial interests/personal relationships which may be considered as potential competing interests:

All authors have completed the ICMJE uniform disclosure form at https://www.icmje.org/disclosure-of-interest/ and declare:

**A.B., A.B.S., V.J., A.W., A.M.** declare no conflicts of interest.

**C.W.** reports grants from British Heart Foundation and National Institute for Health and Care Research and has unpaid roles with *EHJ Quality of Care and Clinical Outcomes* (associate editor), and NICE Indicator Advisory Committee.

**M.H.** reports grants from NIHR, MRC, European Huntington’s Disease Network, UKRI, and Proctor & Gamble.

**T.Me** is a member of the European Society of Cardiology – Regulatory Affairs Committee, European Medicines Agency – medical device expert panel and is chair of the Biomedical Alliance in Europe, Regulatory Affairs Committee.

**T.W.** is the president of the Global Allergy and Airways Patient Platform.

**J.D.** and **M.R.** are members of the European Society of Cardiology Patient Forum.

**C.P.G**. has received grants from Alan Turing Institute, British Heart Foundation, National Institute for Health and Care Research, Horizon 2020, Abbott Diabetes, Bristol Myers Squibb and European Society of Cardiology and reports consulting fees from AI Nexus, AstraZeneca, Amgen, Bayer, Bristol Myers Squibb, Boehrinher-Ingleheim, CardioMatics, Chiesi, Daiichi Sankyo, GPRI Research B.V., Menarini, Novartis, iRhythm, Organon and The Phoenix Group and speaker’s fees from AstraZeneca, Boston Scientific, Menarini, Novartis, Raisio Group, Wondr Medical, Zydus. C.P.G. is also a deputy editor: *EHJ Quality of Care and Clinical Outcomes*, NICE Indicator Advisory Committee member, chair ESC Quality Indicator Committee member and participated on a Data Safety Monitoring Board or Advisory Board for DANBLCOK trial and TARGET CTCA trial.

**M.J.C.** is director of the Centre for the Centre for Patient Reported Outcomes Research, deputy director of the Birmingham Health Partners Centre for Regulatory Science and Innovation and is a National Institute for Health and Care Research (NIHR) Senior Investigator. M.J.C. receives funding from the National Institute for Health and Care Research (NIHR), UK Research and Innovation (UKRI), NIHR Birmingham Biomedical Research Centre (BRC), NIHR ARC West Midlands, LifeArc, European Regional Development Fund, Innovate UK (part of UKRI), Merck, GSK and Gilead. M.J.C. has received personal fees from Aparito Ltd, Boehringer Ingelheim, CIS Oncology, Halfloop, ICON, Merck, Pfizer and Vertex outside the submitted work. In addition, a family member owns shares in GSK. She is a member of the PROTEUS Consortium. All other authors declare no support from any organisation for the submitted work; no financial relationships with any organisations that might have an interest in the submitted work in the previous 3 years; no other relationships or activities that could appear to have influenced the submitted work.

**MDM** reports honoraria from AstraZeneca, Janssen, Merck Sharp & Dohme (MSD), Novartis, Pfizer, Roche, GlaxoSmithKline, Amgen, Merck, Takeda, Ipsen, Viatris for consultancy or participation to advisory boards; direct research funding from Tesaro/GlaxoSmithKline; institutional funding for work in clinical trials/contracted research from Beigene, Exelixis, MSD, Pfizer and Roche.

**AR** has received research grants from NIHR, CIHR, Horizon 2020, AO UK&I, DePuty J&J Ltd, and Stryker Ltd. He is a member of the NIHR i4i funding board.

## Data Availability

All data requests should be submitted to c.p.gale@leeds.ac.uk for consideration.
