# Supplementary material for: A FeAsibility items Checklist for assessing implementation characTeristics of patient-reported Outcome measures in Research, Regulation and Routine clinical care (FACTOR3): Development and evaluation
Source: Clin Med (Lond). 2026 May 27;26(4):100592. doi: 10.1016/j.clinme.2026.100592 (PMC13315201; doi:10.1016/j.clinme.2026.100592)
Supplement: Supplementary file 1 — Supplementary material [file mmc1.docx]

**Supplementary Section:**

**Search Strategy**

Search strategy for scoping review included the following terms:

“Patient reported outcome measure” OR “Patient reported outcome” OR “PROM” OR “PRO” AND “Feasibility” OR “Feasible” OR “Practical” AND “Implementation” OR “Implement” OR “Barrier”

**Table 1:** Evolution of FACTOR3 items

**Table 2:** Each reviewer’s score for each PROM.

| **PROM** | **Reviewer 1** | **Reviewer 2** | **Reviewer 3** |
| --- | --- | --- | --- |
| **EQ5D-5L** | 8 | 8 | 8 |
| **Oxford Hip Score** | 8 | 8 | 8 |
| **Re-QOL-10** | 7 | 6 | 6 |
| **EORTC-QLQ-30** | 5 | 3 | 4 |
| **HeartQOL** | 5 | 5 | 5 |
| **NEI VFQ-25** | 7 | 4 | 5 |

**Table 3**: FACTOR3 checklist table.

1. Is it free to use according to the intended context?
2. Is a license required?
3. Are the questions easily understood?
4. Does the duration of PROM align with the goal?
5. Does the PROM cover all necessary aspects of the disease (or regulatory approval for trials only)?
6. Are there culturally validated translations of the PROM in the desired language(s)?
7. Is the PROM compatible with electronic devices?
8. Does the PROM contain minimal important difference?

Yes answers equate to a score of 1 and No equates to 0.
